# Macrolide-Resistant *Mycoplasma pneumoniae* Infection, Japan, 2008–2015

**DOI:** 10.3201/eid2310.170106

**Published:** 2017-10

**Authors:** Takaaki Tanaka, Tomohiro Oishi, Ippei Miyata, Shoko Wakabayashi, Mina Kono, Sahoko Ono, Atsushi Kato, Yoko Fukuda, Aki Saito, Eisuke Kondo, Hideto Teranishi, Yuhei Tanaka, Tokio Wakabayashi, Hiroto Akaike, Satoko Ogita, Naoki Ohno, Takashi Nakano, Kihei Terada, Kazunobu Ouchi

**Affiliations:** Kawasaki Medical School, Okayama, Japan

**Keywords:** Mycoplasma pneumoniae, antimicrobial resistance, macrolides, drug resistance, Japan, children, real-time PCR, children, bacteria

## Abstract

We evaluated isolates obtained from children with *Mycoplasma pneumoniae* infection throughout Japan during 2008–2015. The highest prevalence of macrolide-resistant *M. pneumoniae* was 81.6% in 2012, followed by 59.3% in 2014 and 43.6% in 2015. The prevalence of macrolide-resistant *M. pneumoniae* among children in Japan has decreased.

Since the early 2000s, macrolide-resistant *Mycoplasma pneumoniae* isolates have been identified in Japan. We previously reported the results of our national surveillance study that investigated the prevalence of macrolide-resistant *M. pneumoniae* infection among children in Japan during 2008–2012 (*1*). An *M. pneumoniae* pandemic occurred in Japan during 2010–2012, especially among children. Similar pandemics also occurred in other countries (*2*). However, the prevalence of macrolide-resistant *M. pneumoniae* infection also gradually increased at the same time in Asia, including Japan (*1**,**2*). We investigated the prevalence of macrolide-resistant *M. pneumoniae* infection after the pandemic.

## The Study

Children with respiratory tract infections who visited 68 medical institutions in Japan were classified according to district: 1) Kyushu (population 14 million); 2) Chugoku-Shikoku (11 million); 3) Kinki (20 million); 4) Kanto-Chubu (62 million); and 5) Tohoku-Hokkaido (14 million) (Figure). Most patients had been enrolled in the Atypical Pathogen Study Group before the 2010–2012 pandemic; however, some did not participate in our previous study reported in 2013 (*1*). Here, we report the data for January 2008–December 2015.

**Figure:**
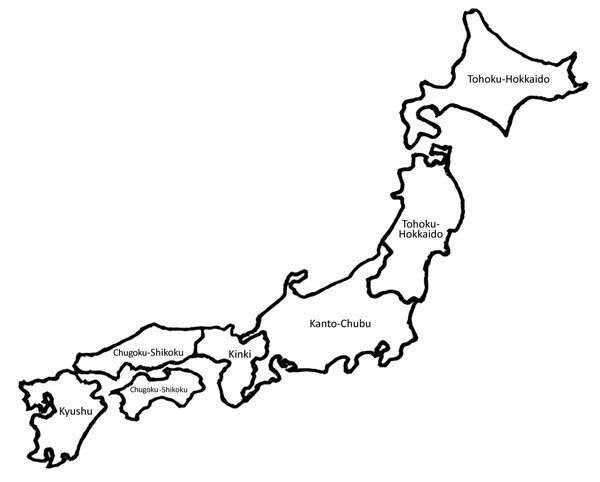
Districts of origin for patients with *Mycoplasma pneumoniae* infection, Japan, 2008–2015: 1, Kyushu; 2, Chugoku-Shikoku; 3, Kinki; 4, Kanto-Chubu; 5, Tohoku-Hokkaido.

As previously reported (*1*), pediatricians collected nasopharyngeal swab samples and sputum samples, when available, from children with respiratory tract infections. Participants’ parents provided informed consent before sample collection. The Ethics Committee of the Kawasaki Medical School (Kurashiki, Japan) approved the study protocol on December 8, 2014 (no. 286–3).

Nasopharyngeal swab specimens were collected with a sterile swab (Nippon Menbo, Saitama, Japan). After collection, each swab was placed into 3 mL of Universal Vial Transport Medium (Becton, Dickinson and Company, Sparks, MD, USA) and transported at room temperature within 2 days to Kawasaki Medical School by a parcel delivery system. Crude DNA extracts were obtained with the following procedure: 300 μL of resuspended transport medium was transferred into a 1.5-mL microtube centrifuged at 4°C, 20,000 × *g* for 30 min, after which 285 μL of supernatant was discarded; the remainder was transferred into a thin-wall 200-μL PCR tube after resuspension with 85 μL lysis buffer by gentle pipetting. This suspension was incubated at 55°C for 60 min, followed by 100°C for 10 min before cooling to 4°C. The composition of the lysis buffer was Tris-HCl (pH 8.3) 2 mmol/L, KCl 10 mM, MgCl_2_ 0.045 mM, Triton X-100 0.45 o/o, Tween 20 0.45%, and RNA-grade Proteinase K (Thermo Fisher Scientific Inc., Waltham, MA, USA) 0.4 µg/µL. *M. pneumoniae* DNA was detected by real-time PCR targeting a conserved part of the gene coding for the P1 adhesin (*3*). 

We searched for mutations at sites 2063, 2064, and 2617 in domain V of 23S rRNA of *M. pneumoniae* using a direct sequencing method with isolates or samples with a positive PCR result, as reported previously (*3*). For this study, we investigated 1,448 samples obtained from patients in Japan who had respiratory tract infections; we detected *M. pneumoniae* DNA by real-time PCR and searched for mutations using a direct sequencing method.

The overall prevalence rate of macrolide-resistant *M. pneumoniae* in Japan was 70.2% (1,016/1,448) and ranged from 43.7% in Kyushu to 89.3% in Kanto-Chubu (Table 1). When divided into 3 time periods (prepandemic, pandemic, and postpandemic), the overall rate of macrolide-resistant *M. pneumonia* was substantially lower in the post epidemic period than in the pandemic period; macrolide-resistant *M. pneumoniae* isolates decreased in 4 of the 5 locations.

**Table 1:** *Mycoplasma pneumoniae* infections diagnosed by real-time PCR and prevalence of macrolide-resistant *M. pneumoniae*, by district, Japan

District	Average age, y (range)	No. patients (M:F)	Macrolide resistance, % (no. positive/total no. patients)*			
2008–2010	2011–2012	2013–2015	Total
Kyushu	6.4 (0–14)	239 (1.2:1)	–	64.1 (82/128)	27.9 (31/111)	47.3 (113/239)
Chugoku-Shikoku	7.3 (0–15)	623 (1.4:1)	68.6 (59/86)	70.6 (339/480)	80.7 (46/57)	71.3 (444/623)
Kinki	7.1 (1–15)	227 (1:1)	33.3 (7/21)	86.1 (162/188)	66.7 (12/18)	79.7 (181/227)
Kanto-Chubu	7.5 (0–13)	268 (1.1:1)	–	80.1 (197/246)	72.7 (16/22)	79.5 (213/268)
Hokkaido-Tohoku	7.7 (0–13)	91 (1.2:1)	84.2 (32/38)	68.1 (32/47)	16.7 (1/6)	71.4 (65/91)
Total	7.3 (0–15)	1,448 (1.3:1)	67.6 (98/145)	74.6 (812/1,089)	49.5 (106/214)	70.2 (1,016/1,448)

*2008–2010, prepandemic; 2011–2012, pandemic; 2013–2015, post pandemic.

The peak rate of macrolide-resistant *M. pneumoniae* infection was 81.6% in 2012 (493/604) (Table 2). Rates of macrolide-resistant *M. pneumoniae* infection gradually decreased as follows: 65.8% (25/38) in 2013, 59.3% (16/27) in 2014, and 43.6% (65/149) in 2015. The most frequent mutation was A2063G mutation (95.8%), followed by A2063T (3.1%), A2064G (0.6%), A2063C (0.3%), C2617G (0.2%), and C2617T (0.1%).

**Table 2:** Rates of macrolide-resistant *Mycoplasma pneumoniae* and point mutations in domain V of 23S rRNA, Japan

Variable	2008	2009	2010	2011	2012	2013	2014	2015	Total
Macrolide resistance, % (no. positive/total no. patients)	55.6 (10/18)	72.7 (8/11)	69.0 (80/116)	65.8 (319/485)	81.6 (493/604)	65.8 (25/38)	59.3 (16/27)	43.6 (65/149)	70.2 (1,016/ 1,448)
Point mutations, no. (%)									
A2063G	10 (100)	8 (100)	80 (100)	301 (94.4)	471 (95.5)	25 (100)	16 (100)	62 (95.2)	97.3 (95.8)
A2063C	0	0	0	3 (0.9)	0	0	0	0	3 (0.3)
A2063T	0	0	0	15 (4.7)	14 (2.3)	0	0	2(3.2)	31 (3.1)
A2064G	0	0	0	0	6 (1.0)	0	0	0	6 (0.6)
C2617G	0	0	0	0	2 (0.3)	0	0	0	2 (0.2)
C2617T	0	0	0	0	0	0	0	1 (1.6)	1 (0.1)

## Conclusions

The prevalence of macrolide-resistant *M. pneumoniae* infection was high during 2008–2012 but gradually decreased throughout Japan during 2013–2015. One reason for this decrease might be the 2011 publication of guidelines for treating *M. pneumoniae* pneumonia (*4*). Because of the higher prevalence of macrolide-resistant *M. pneumoniae* infection in children than in adults (5), respiratory fluoroquinolone/tosufloxacin was recommended for use in patients in whom *M. pneumoniae* pneumonia responded poorly to macrolide treatment in these guidelines. After the pandemic, the guideline committee addressed the concerns of further accumulation of macrolide-resistant *M. pneumoniae* in children resulting from constant macrolide use and teeth damage from tetracycline use in children <8 years of age (*4*). Tosufloxacin was approved for use in children in 2010 in Japan and has been used to treat *M. pneumoniae* infection. Because the guidelines recommend tosufloxacin as a second-line drug, pediatricians in Japan may be using the appropriate antimicrobial drugs for *M. pneumoniae* infection in accordance with these guidelines, which might have led to the decrease in incidence. Unfortunately, to our knowledge, no reports have been published to support these hypotheses. However, prescriptions for oral antimicrobial drugs in Japan comprise most of the prescriptions for antimicrobial drugs (*6*); therefore, we believe that the rate of macrolide-resistant *M. pneumoniae* might be affected by changes in the use of oral macrolide agents.

In addition, the prevalence of macrolide-resistant *M. pneumoniae* infection varies among countries: for example, 13.2% in the United States (*7*), 8.3% in France (*8*), and 3.1% in Germany (*9*). These variations might be attributed to differences in the number of prescription macrolide agents among countries. Although accurately assuming the number of prescription macrolide agents in each country is difficult, we can estimate the macrolide resistance rate of *Streptococcus pneumoniae* among those countries. A recent report supported the hypothesis that antimicrobial selection pressure results in clonal expansion of existing resistant strains (*10*). In Japan and China, which have a high prevalence of macrolide-resistant *M. pneumoniae* (*3**,**5*), the rates of macrolide-resistant *S. pneumoniae* also are very high (*11**,**12*). In countries with low rates of macrolide-resistant *M. pneumoniae*, such as the United States, France, and Germany (*7**,**8**,**9*), the prevalence rates of macrolide-resistant *S. pneumoniae* are low (*13**,**14*). However, the mechanisms of macrolide resistance are difficult to compare between *Streptococcus* and *Mycoplasma* (*15*). Therefore, we also interviewed the pediatricians who collected the samples for this study, and analysis of their responses is ongoing. Upon completion of these interviews, we will be able to report patients’ background characteristics, such as previous use of macrolides and medical examination histories; this information may provide further insight into the decreased prevalence of macrolide-resistant *M. pneumoniae* infection.

In summary, the prevalence of macrolide-resistant *M. pneumoniae* infection in children in Japan was high and increased between 2008 and 2012 but declined thereafter. Careful continuous monitoring of macrolide-resistant *M. pneumoniae* infection rates in Japan and other countries is needed.
